# Direct estimate of the internal π-donation to the carbene centre within N-heterocyclic carbenes and related molecules

**DOI:** 10.3762/bjoc.11.294

**Published:** 2015-12-24

**Authors:** Diego M Andrada, Nicole Holzmann, Thomas Hamadi, Gernot Frenking

**Affiliations:** 1 Fachbereich Chemie, Philipps-Universität Marburg, Hans-Meerwein-Strasse, D-35032 Marburg, Germany; 2 Laboratoire International Associé Centre National de la Recherche Scientifique - UMR 7565, Université de Lorraine, 54506 Vandoeuvre-lès-Nancy, France

**Keywords:** bonding analysis, N-heterocyclic carbenes, π-donation

## Abstract

Fifteen cyclic and acylic carbenes have been calculated with density functional theory at the BP86/def2-TZVPP level. The strength of the internal X→p(π) π-donation of heteroatoms and carbon which are bonded to the C(II) atom is estimated with the help of NBO calculations and with an energy decomposition analysis. The investigated molecules include N-heterocyclic carbenes (NHCs), the cyclic alkyl(amino)carbene (cAAC), mesoionic carbenes and ylide-stabilized carbenes. The bonding analysis suggests that the carbene centre in cAAC and in diamidocarbene have the weakest X→p(π) π-donation while mesoionic carbenes possess the strongest π-donation.

## Introduction

Since the isolation and unambiguous characterization of imidazol-2-ylidene by Arduengo in 1991 [1], the chemistry of stable singlet carbenes has become a major field of chemical research [2–4]. The outstanding stability and synthetic utility of N-heterocyclic carbenes (NHCs) is an ongoing subject to an ubiquitous number of experimental and computational studies exploring their structural and electronic properties [5–8]. In the last two decades, these versatile compounds have been widely employed in transition metal [9–13] and organocatalysis [14–16], organometallic [17–19] and main group synthesis [20–26], and activation of small molecules [27–28].

NHCs possess a divalent C(II) atom which is connected to one or two nitrogen atoms [2,29]. The adjacent heteroatoms stabilize the singlet form by both their σ-electron-withdrawing character and the π-electron-donation of their lone pairs into the formally empty 2p orbital of C(II), giving rise to a four π-electron three-centre system [1–2,30–31]. Thus, the lone pair placed in the plane of the ring renders NHCs as nucleophilic compounds while the partially empty 2p orbital on C_carb_ provides some π-acceptor character. It has been initially claimed that the excellent ligand features of NHCs were due to their strong σ-donation abilities [32–33]. However, experimental and computational evidences have revealed non-negligible π-acceptor properties [34–38]. In recent years, several strategies have successfully been developed for tuning the π-acidity of NHCs by changing substitution and structural patterns, such as the size of the backbone ring [39–41], variation of the α-heteroatoms [7], anti-Bredt NHCs [42–43], mesoionic NHCs [44–47], ylide stabilized carbenes [48–51] and other [52–55]. A remarkable variation was introduced with the cyclic alkyl(amino)carbene (cAAC) by Bertrand in 2005 [20,56–57]. The replacement of one amino substituent by a saturated alkyl group makes the carbene more nucleophilic and electrophilic at the same time [20,56–57]. Since then, cAACs have been used as a superior ligand for the stabilization of unstable chemical species, radical and main group elements in different oxidation states [27,34,36,58–60], due to their stronger π-acceptor and σ-donor properties.

With such a wide range of NHCs, a thorough knowledge of the electronic nature is a prerequisite for a guided design of suitable applications. In this regard, a number of techniques have been developed to quantify the π-acceptor ability of carbenes [61–62]. Thus, NMR methods have been reported that allow the measurement of the π-acidity of NHCs [63]. Bertrand et al. and Ganter et al. have proposed the use of ^31^P and ^77^Se NMR chemical shift of the NHC-phenylphosphinidene and NHC-selenium adducts, respectively, to determine the π-acceptor strength of the parent NHCs [64–65]. In the same way, Nolan et al. have applied this technique to a wider range of NHCs and have established the connection between the π-accepting abilities and the NMR chemical shift [66]. Furthermore, different theoretical approaches can be found in the literature where natural bond orbital calculations (NBO) and energy decomposition analysis (EDA) have been applied to a broad variety of organometallic complexes [35,67–72]. Although all the procedures have proven to be a convenient way to evaluate the π-acceptor capacities of NHCs, they are limited by the fact that they inherently estimate properties of the parents systems after complexation. It would be helpful if the intrinsic π-donor strength of the substituents to the carbene centre would be directly estimated in the parent carbenes.

**Scheme 1:**
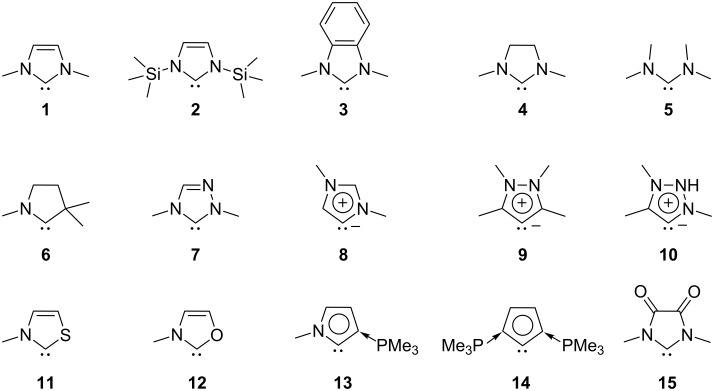
Schematic view of the calculated carbenes **1–15**.

In the quest of a direct estimate of the NHC π-acceptor properties and its connection with the π-stabilization exerted by the adjacent α-heteroatoms to the carbene carbon atom, herein we report on the use of the EDA-NOCV (energy decomposition analysis with natural orbitals for chemical valence) method to evaluate the intrinsic electronic π-donation strength. In this context, we quantitatively estimate the differences in the electronic structure of 15 archetypical carbenes (Scheme 1). Here compounds **1**–**4**, **6**, and **7** are typical NHCs while **5** is an acylic diamidocarbene. Compounds **8**–**10** are so-called abnormal or mesoionic carbenes for which no resonance form without formal charges can be written [73]. Molecules **11** and **12** are NHCs with one nitrogen donor atom where the carbene centre is additionally stabilised by another hetero π-donor. Compounds **13** and **14** are ylide-stabilised carbenes while **15** is a diamidocarbene.

## Computational Details

All geometries were optimized without symmetry constraint within the DFT framework using the BP86 functional [74–75] in combination with the Gaussian basis sets def2-TZVPP [76]. Stationary points were located with the Berny algorithm [77] using redundant coordinates. Analytical Hessians were computed to determinate the nature of the stationary points [78]. All geometry optimization computations were performed using the Gaussian 09 suite of programs [79]. Wiberg Bond Orders [80] and NPA [81–82] atomic partial charges have been calculated at the BP86/def2-TZVPP [74–76] level of theory with GAUSSIAN 09 [79] and GENNBO 5.9 programs [83].

All energy decomposition analyses were carried out using the BP86 functional in combination with uncontracted Slater-type orbitals (STOs) as basis function for the SCF calculations [84]. The basis sets for all elements were triple-ζ quality augmented by two sets of polarizations functions and one set of diffuse functions. Core electrons were treated by the frozen-core approximation. This level of theory is denoted as BP86/TZ2P+. We did not reoptimize the geometries but used the BP86/def2-TZVPP optimized structures, because we know from previous studies that the two basis sets give very similar geometries. An auxiliary set of s, p, d, f, and g STOs was used to fit the molecular densities and to represent the Coulomb and exchange potentials accurately in each SCF cycle [85]. Scalar relativistic effects have been incorporated by applying the zeroth-order regular approximation (ZORA) [86]. The nature of the stationary points on the potential energy surface was determined by calculating the vibrational frequencies at BP86/TZ2P+. These calculations were performed with the program package ADF.2013 [87].

The bonding situation of the donor–acceptor bonds was investigated by an energy decomposition analysis (EDA) which was developed by Morokuma [88] and by Ziegler and Rauk [89–90]. The bonding analysis focuses on the instantaneous interaction energy ∆*E*_int_ of a bond A–B between two fragments A and B in the particular electronic reference state and in the frozen geometry AB. This energy is divided into three main components (Equation 1).

[1]
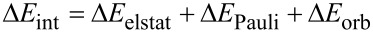


The term ∆*E*_elstat_ corresponds to the classical electrostatic interaction between the unperturbed charge distributions of the prepared atoms (or fragments) and it is usually attractive. The Pauli repulsion ∆*E*_Pauli_ is the energy change associated with the transformation from the superposition of the unperturbed wave functions of the isolated fragments to the wave function Ψ^0^ = NÂ[Ψ_A_Ψ_B_], which properly obeys the Pauli principle through explicit antisymmetrization (Â operator) and renormalization (N = constant) of the product wave function. It comprises the destabilizing interactions between electrons of the same spin on either fragment. The orbital interaction ∆*E*_orb_ accounts for charge transfer and polarization effects [91]. The ∆*E*_orb_ term can be dissected into contributions from each irreducible representation of the point group of the interacting system. Further details on the EDA method and its applications to the analysis of the chemical bond [92–94] can be found in the literature.

The EDA with natural orbitals for chemical valence (EDA-NOCV) method [95] combines charge and energy decomposition schemes to split the deformation density which is associated with the bond formation, ∆ρ, into different components of the chemical bond. The EDA-NOCV calculations provide pairwise energy contributions for each pair of interaction orbitals to the total bond energy. NOCV is defined as the eigenvector of the valence operator, 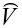, given by Equation 2 [96–98].

[2]
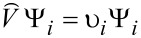


In the EDA-NOCV scheme the orbital interaction term, ∆*E*_orb_, is given by Equation 3.

[3]
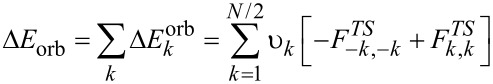


Where 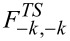 and 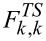 are diagonal Kohn–Sham matrix elements corresponding to NOCVs with the eigenvalues –ν_k_ and ν_k_, respectively. The 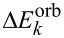 terms are assigned to a particular type of bond by visual inspection of the shape of the deformation density, ∆ρ_k_. The absolute values │ν_k_│ of the eigenvalues of Equation 3 give the charge flow which is associated with each pairwise orbital interaction. The EDA-NOCV scheme thus provides information about the charge deformation (∆ρ_orb_) and the associated stabilization energy (∆*E*_orb_) of the orbital interactions in chemical bonds. For more details we refer to the literature [97–98].

## Results and Discussion

**Figure 1:**
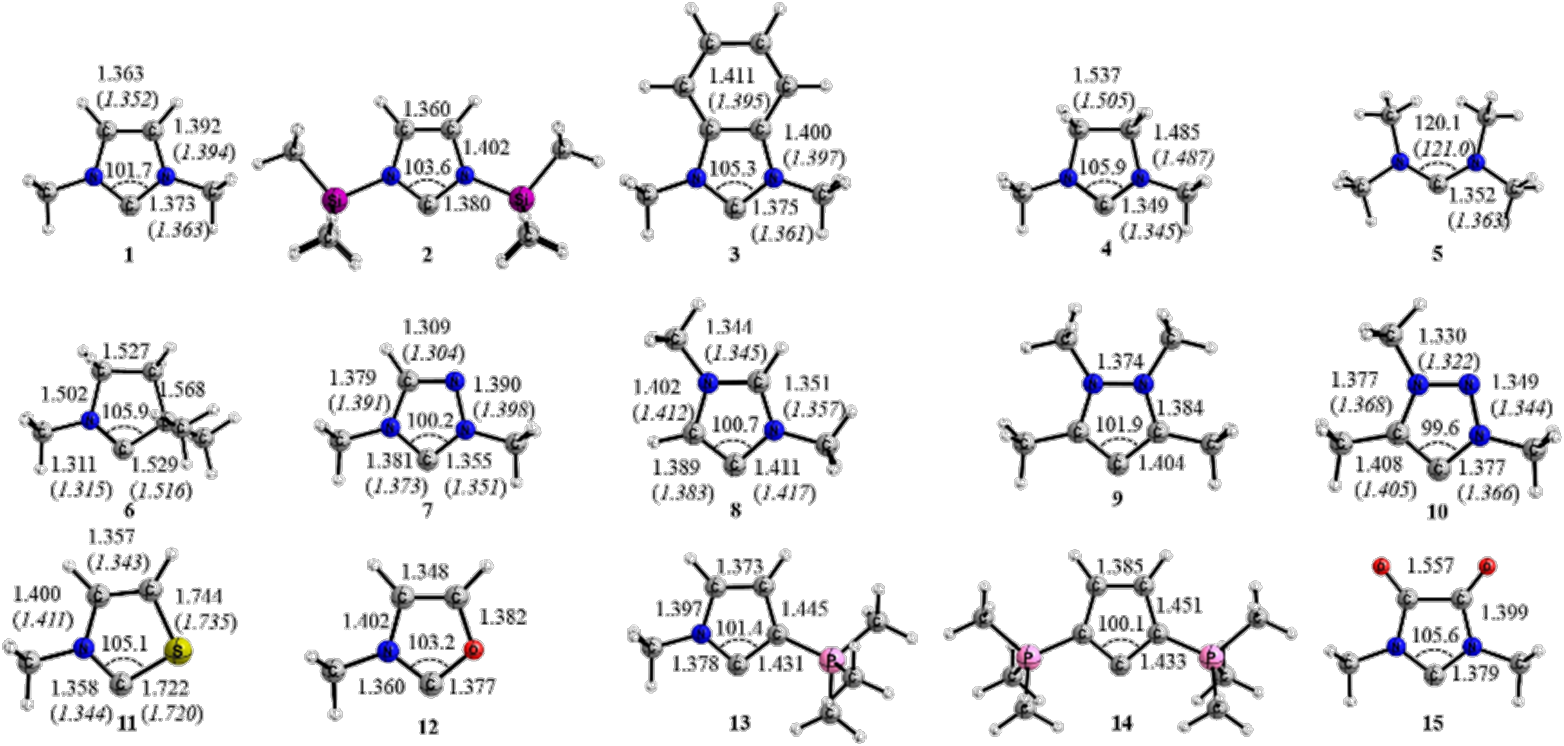
Optimized geometries of carbenes **1–15** at the BP86/def2-TZVPP level of theory. Bond lengths and angles are given in [Å] and [°]. Experimental values are given in parentheses: **1** (Ref. [31]); **3** (Ref. [99]); **4** (Ref. [100]); **5** (Ref. [101]); **6** (Ref. [56]); **7** (Ref. [102]); **8** (Ref. [46]); **10** (Ref. [47]); **11** (Ref. [103]).

The optimized geometries at the BP86/def2-TZVPP level of theory of the calculated carbenes and the most important bond lengths and angles are shown in Figure 1. Experimental values of substituted analogues are given in parentheses.

The theoretically predicted structures are in good agreement with experimental data [31,46–47,56,99–103]. In general the computed bond lengths are slightly longer than the experimental ones. The X–C_carb_–X (X = N, C, O and S) angle in the five-membered rings slightly varies between 99.6° (**10**) and 105.6° (**15**) and is slightly larger (120.1°) in the acyclic carbene **5**. This angle is often associated with the σ-donor properties which are related to the sp^x^ hybridization of the carbene lone pair orbital [2]. A first hint of the strength of the π-donation is given by the C_carb_–N bond lengths. The shortest C_carb_–N bond of 1.311 Å is calculated for the cAAC species **6**, which is close to a standard C=N double bond (1.30 Å), while the longest value of 1.411 Å is calculated for the abnormal carbene **8**, which approaches a standard C–N single bond (1.46 Å) [104]. The C_carb_–N bond lengths exhibit otherwise a remarkable small range between 1.35–1.38 Å. The C–N bond is slightly longer in the conjugated 6π-electron carbenes which possess some aromatic character than in the non-aromatic analogues which previously ascribed to stronger π-conjugation [105–107]. The introduction of the heteroatoms O and S in compounds **11** and **12** changes the C–N bond only slightly. The C_carb_–C bond lengths in the conjugated carbenes are between 1.404 Å (**9**)–1.433 Å (**14**) while the cAAC system **6** has a much longer distance of 1.529 Å.

**Figure 2:**
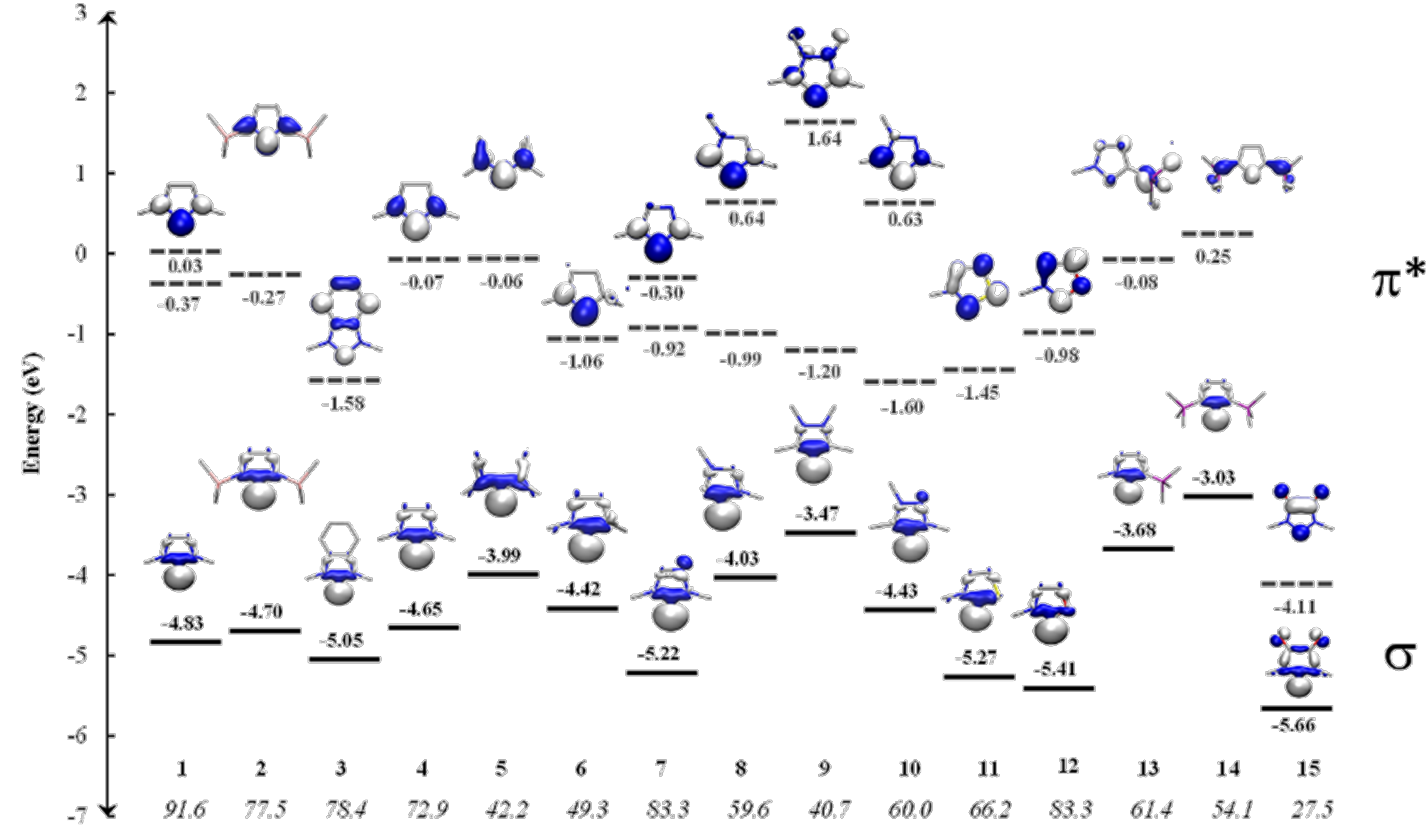
Frontier orbitals (BP86/def2-TZVPP) and eigenvalues (in eV) of the carbenes **1–15**. The isosurfaces were taken at the 0.06 isovalue. The hydrogen atoms are omitted for clarity.

Figure 2 shows the shape and energy of the frontier molecular orbitals for compounds **1–15** which are relevant for the σ-donor and π-acceptor properties. The HOMO is in all cases a carbon σ-lone pair while the LUMO (LUMO + 1 for **1**, **7–10**) depicts a π-orbital which has the largest coefficient at the C_carb_ atom that makes it suitable for π-backdonation. The LUMOs of compounds **1**, and **7–10** which are not displayed in Figure 2 are also π-orbitals which have a node at the C_carb_ atom. The HOMO–LUMO energy difference varies considerably between 4.58 eV (**4**) and 1.55 eV (**15**). The small HOMO–LUMO gap of the diamidocarbene **15** comes from the very low lying LUMO which has been noted before [42]. There is clearly a correlation between the HOMO–LUMO energy difference and the calculated singlet–triplet gap of the compounds which are given at the bottom of Figure 2. The largest singlet–triplet (S/T) gap is predicted for compound **1** (91.6 kcal/mol) while compound **15** possesses the lowest S/T value (27.5 kcal/mol).

The focus of the present work lies on the π-donation from the neighboring atoms to the carbene center X(π)→C_carb_. To estimate the size of the charge donation Δq(π) we calculated the occupation of p(π) AO of the carbene atom in molecules **1**–**15** which is available from the NBO analysis. Table 1 gives the computed values for the atomic charges, the orbital occupation of the σ-lone pair orbital and the occupation of p(π) AO of the C_carb_ atom. We also present the Wiberg Bond Orders for the C_carb_−X bonds.

**Table 1:** Calculated NBO partial charges q(C_carb_) of the carbene carbon atom, occupation of the lone pair orbital C_carb_(σ) and the p(π) AO at C_carb_. Wiberg Bond Orders (WBO) for the C_carb_–X (X = C, N, O and S) bonds at BP86/def2-TZVPP.

	q(C_carb_)	C_carb_(σ)	p(π)	WBO

**1**	0.04	1.91	0.69	1.27
**2**	0.06	1.88	0.67	1.29
**3**	0.09	1.91	0.64	1.25
**4**	0.13	1.86	0.60	1.32
**5**	0.12	1.84	0.62	1.34
**6**	0.09	1.87	0.49	1.56/1.00^a^
**7**	0.05	1.90	0.67	1.22/1.35^a^
**8**	−0.19	1.88	0.81	1.14/1.60^a^
**9**	−0.39	1.83	0.81	1.46
**10**	−0.17	1.88	0.73	1.44/1.26^a^
**11**	−0.23	1.89	0.73	1.34/1.38^a^
**12**	0.19	1.91	0.63	1.33/1.13^a^
**13**	−0.16	1.88	0.74	1.25/1.37^a^
**14**	−0.37	1.85	0.80	1.36
**15**	0.19	1.93	0.51	1.20

^a^The first value is for the atom on the left side of C_carb_ as shown in Figure 1.

The NBO data indicate that the occupation of the p(π) AO of the C_carb_ atom is between 0.81 e (**8**, and **9**) and 0.49 e (**6**). The p(π) occupation at the C_carb_ atom is particularly large for the carbenes which have no heteroatoms bonded to it (**9**, and **14**) or only one heteroatom as in **8**. The special role of the cAAC species **6** which exhibits particular reactivity [52–55] that has recently been utilized for the stabilization of unusual compounds [108–117] comes to the fore by the smallest value of the p(π) occupation. Carbene **6** has also the largest bond order for the C_carb_–N bond and the smallest bond order for a C_carb_–C bond. Note that the C_carb_ atom carries a negative partial charge when it is bonded to one or two carbon atoms (**8**–**11**, **13**, and **14**).

**Scheme 2:**
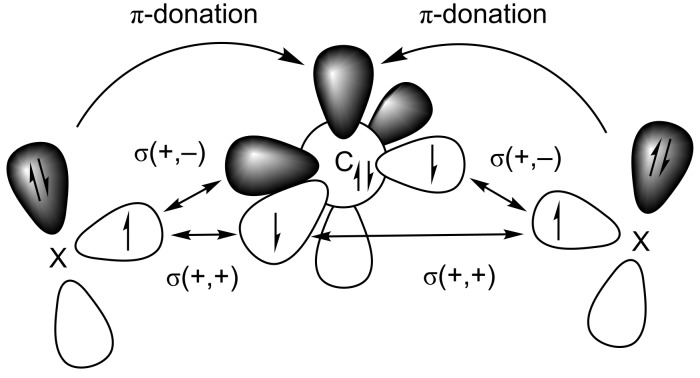
Schematic view of the major orbital interactions between a carbon atom in the ^3^P electronic ground state with the configuration 2s^2^2p_σ_^1^2p_||_^1^2p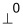 and atoms X which possess a p(π) lone pair orbital. There are σ(+,+) and σ(+,−) interactions which give the two C_carb_–X σ-bonds and the π-donation X(π)→C_carb_.

The energy contribution of the X(π)→C_carb_ donation can be calculated with the EDA-NOCV method which is described in the method section. We carried out EDA-NOCV calculations using a carbon atom in the ^3^P ground state with the electronic configuration 2s^2^2p_σ_^1^2p_||_^1^2p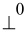 and the remaining fragment as interacting moieties with unpaired electrons at X. Scheme 2 shows the directly interacting atoms C_carb_ and X where the electrons are placed in such a way that the unpaired electrons on both fragments are in the plane of the molecule yielding the σ-bonds while the lone pair electrons and the vacant p AO of carbon have π-symmetry with respect to the molecular plane. This leads to three major orbital interactions for σ and π-bonding between C_carb_ and X. These are the σ(+,+) and σ(+,−) interactions that come from the in-phase and out-of-phase combinations of the lone-pairs, respectively, which give the two C_carb_–X σ-bonds and the π-donation X(π)→C_carb_.

Table 2 shows the numerical results of the EDA-NOCV calculations. The total interaction energy Δ*E*_int_ between the carbon atom and the remaining fragment in the frozen geometry [118] is composed from the stabilizing orbital (covalent) interactions Δ*E*_orb_ and the Coulombic term Δ*E*_elstat_ and the destabilizing Pauli repulsion Δ*E*_Pauli_. The strongest attraction comes from the orbital term Δ*E*_orb_. We want to point out that the trend of the intrinsic bond strength between C_carb_ and the remaining fragment does not correlate with the trend of covalent bonding. The largest Δ*E*_int_ values are calculated for compounds **8** and **9** but the Δ*E*_orb_ values of the two species are much smaller than those of most other carbenes. The strong net bonding in **8** and **9** is rather related to the comparatively weak Pauli repulsion Δ*E*_Pauli_ which is much weaker than in most other species (Table 2). The interplay of all three factors Δ*E*_orb_, Δ*E*_Pauli_ and Δ*E*_elstat_ for determining the overall net strength of chemical bonding has been highlighted before [92–94,119–120].

**Table 2:** EDA-NOCV calculations at the BP86/TZ2P+ level of theory of compounds **1–15** using C(II) in the valence configuration 2s^2^2p_σ_^1^2p_||_^1^ 2p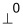 and the remaining fragment as interacting moieties^a^. Energy values are given in kcal/mol.

	**1**	**2**	**3**	**4**^b^	**5**^b^	**6**	**7**	**8**

∆*E*_int_	−322.7	−295.2	−309.6	−319.5	−314.5	−272.3	−302.1	−330.85
∆*E*_Pauli_	759.2	825.7	807.5	804.1	814.41	734.7	846.2	669.18
∆*E*_elstat_^a^	−397.8 (36.8%)	−415.2 (37.1%)	−413.4 (37.0%)	−416.5 (37.1%)	−414.6 (36.7%)	−387.1 (38.4%)	−426.5 (37.2%)	−371.9 (37.2%)
∆*E*_orb_^a^	−684.0 (63.2%)	−705.7 (62.9%)	−703.7 (63.0%)	−707.1 (62.9%)	−714.3 (63.3%)	−619.9 (61.6%)	−721.7 (62.9%)	−628.2 (62.8%)
∆*E*_σ (+,−)_^c^	−319.3 (46.7%)	−331.1 (46.9%)	−322.1 (45.8%)	−335.9 (47.5%)	−353.4 (49.5%)	−331.7 (53.5%)	−338.7 (46.9%)	−327.6 (52.1%)
∆*E*_σ (+,+)_^c^	−233.8 (34.2%)	−247.2 (35.0%)	−252.2 (35.8%)	−242.3 (34.3%)	−223.2 (31.2%)	−182.6 (29.5%)	−241.7 (33.5%)	−170.8 (27.2%)
∆*E*_π-donation_^c^	−**93.4** (13.6%)	−**87.8** (12.4%)	−**89.3** (12.7%)	−**86.1** (12.2%)	−**90.2** (12.6%)	−**72.2** (11.6%)	−**92.6** (12.8%)	−**101.3** (16.1%)
∆*E*_rest_^c^	−37.5 (5.5%)	−39.6 (5.6%)	−40.1 (5.7%)	−42.8 (6.1%)	−47.5 (6.6%)	−33.4 (5.4%)	−48.7 (6.7%)	−28.5 (4.5%)

	**9**^b^	**10**	**11**	**12**	**13**	**14**	**15**	

∆*E*_int_	−336.5	−308.9	−263.9	−285.9	−312.1	−301.6	−280.6	
∆*E*_Pauli_	643.5	760.5	715.5	830.2	705.4	660.0	805.6	
∆*E*_elstat_^a^	−377.3 (38.5%)	−406.3 (38.0%)	−378.1 (38.6%)	−397.5 (35.6%)	−380.8 (37.4%)	−365.2 (38.0%)	−406.6 (37.4%)	
∆*E*_orb_^a^	−602.6 (61.5%)	−663.1 (62.0%)	−601.4 (61.4%)	−718.5 (65.4%)	−636.7 (62.6%)	−596.4 (62.0%)	−679.6 (62.6%)	
∆*E*_σ (+,−)_^c^	−285.9 (47.4%)	−335.8 (50.6%)	−256.9 (42.7%)	−361.0 (50.2%)	−326.8 (51.3%)	−283.0 (47.5%)	−322.0 (47.4%)	
∆*E*_σ (+,+)_^c^	−178.5 (29.6%)	−186.6 (28.1%)	−219.0 (36.4%)	−235.7 (32.8%)	−180.9 (28.4%)	−184.9 (31.0%)	−242.0 (35.6%)	
∆*E*_π-donation_^c^	−**109.9** (18.2%)	−**100.6** (15.2%)	−**90.0** (15.0%)	−**82.4** (11.5%)	−**96.4** (15.1%)	−**100.8** (16.9%)	−**74.0** (10.9%)	
∆*E*_rest_^c^	−28.3 (4.6%)	−40.2 (6.1%)	−35.4 (5.9%)	−39.1 (5.4%)	−32.7 (5.1%)	−27.7 (4.6%)	−41.6 (6.1%)	

^a^The values in parentheses give the percentage contribution to the total attractive interactions Δ*E*_elstat_ + Δ*E*_orb_. ^b^The symmetry C_s_ was enforced. ^c^The values in parentheses give the percentage contribution to the total orbital interactions Δ*E*_orb_.

**Figure 3:**
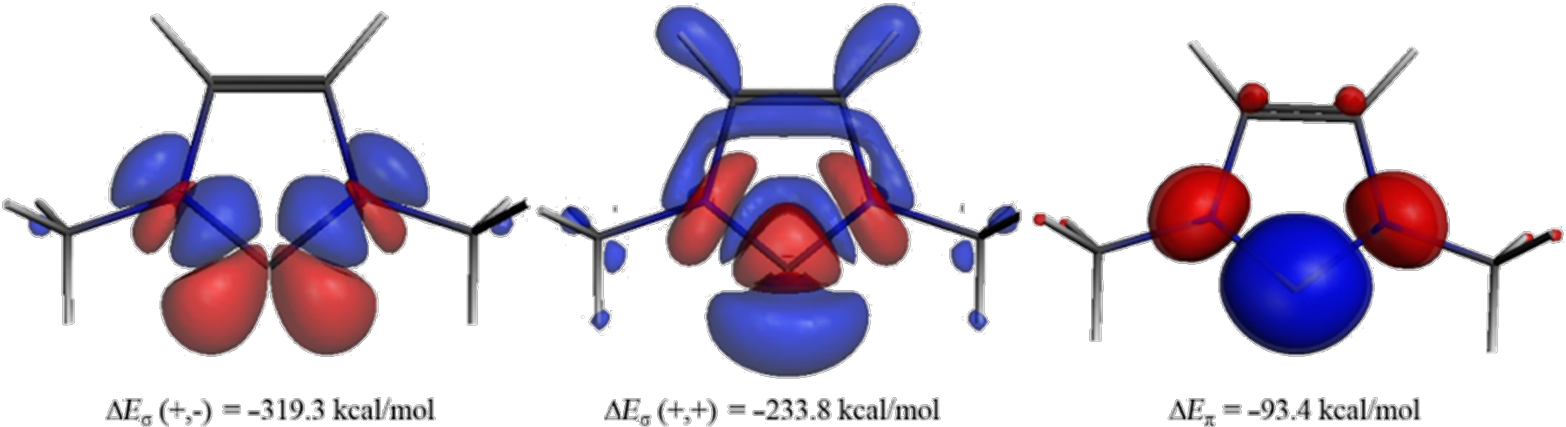
Plot of deformation densities ∆ρ of the pairwise orbital interactions between C(^3^P) and N(Me)HC=CHN(Me), associated energies ∆*E* in kcal/mol. The color-code of the charge flow is red→blue.

The most important information of the EDA-NOCV calculations comes from contributions of the pairwise orbital interactions to Δ*E*_orb_. Table 2 shows that there are indeed three major terms for each molecule which can easily be identified with the schematic description that is given in Scheme 2. The deformation densities associated with the three major orbital interactions ∆*E*_σ_ (+,−), ∆*E*_σ_ (+,+) and ∆*E*_π_ for compound **1** are shown in Figure 3. The color code of the charge deformation on bond formation is red→blue. The largest contributions comes from the formation of the C_carb_–X σ-bonds while the π-donation X(π)→C_carb_ is much weaker which is reasonable. The ∆*E*_σ_ (+,−) component of the C_carb_–N σ-bond (−319.3 kcal/mol, Figure 3a) is bigger than the ∆*E*_σ_ (+,+) component (−233.8 kcal/mol, Figure 3b) which can be explained with the larger overlap of the former term (see Scheme 2). Note that the charge flows of the individual (+,+) and (+,−) interactions have different directions which cannot be easily associated to a physical meaning. It is the net charge flow which indicates the overall direction. The red and blue areas at the carbene carbon in Figure 3b indicates the polarization (change in hybridization) which takes place during the bond formation. The charge flow which is associated with the π-donation X(π)→C_carb_ shows the expected direction from nitrogen to carbon. The charge flow which is associated with the three dominant orbital interactions in compound **2**–**15** is shown in Figure S1 of Supporting Information File 1.

**Figure 4:**
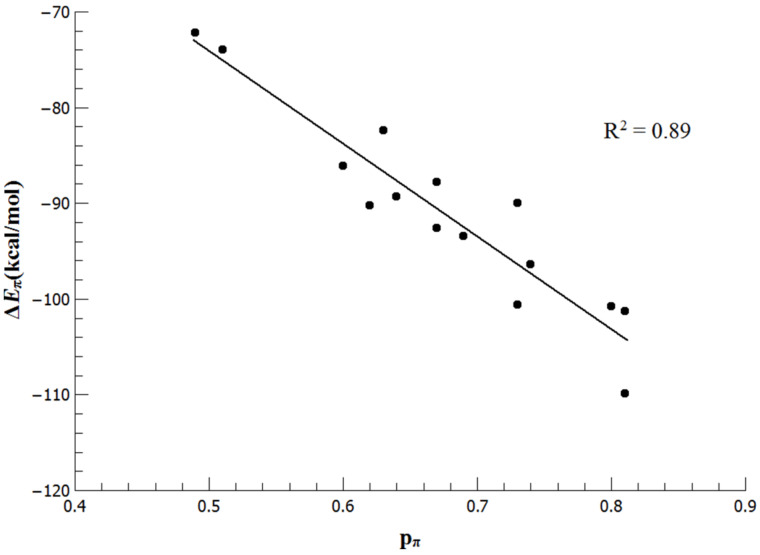
Plot of the Δ*E*_π_ values against NBO p_π_ occupation for the NHC family **1–15**.

Inspection of the strength of ∆*E*_π_ should thus reveal information about the in internal π-donation to the C_carb_ atom in molecules **1**–**15**. Table 2 suggests that the strongest X(π)→C_carb_ donation is found in the 6π-conjugated carbenes **8**–**10**, **13**, and **14** where the C_carb_ atom is bonded to two (**9**, **14**) or one (**8**, **10**, **13**) carbon atoms. The weakest π-donor contributions are calculated for the cAAC species **6** and the diamidocarbene **15**. It appears as if the ∆*E*_π_ values which give the energy contribution of the X(π)→C_carb_ donation which come from the EDA-NOCV calculations and the p(π) occupation which is given by the NBO method correlate. Figure 4 shows a correlation diagram between ∆*E*_π_ and p(π). There is clearly a qualitative correlation between the two entries, but the correlation coefficient of R^2^ = 0.89 indicates that charge donation and associated stabilization of the different systems do not completely agree. Both methods agree that the molecules of cAAC (**6**) and the diamidocarbene **15** possess extremely low π-stabilization of the carbene carbon atom.

## Conclusion

The NBO and EDA-NOCV calculations of the fifteen carbenes show that the carbene centre in cAAC and in diamidocarbene have the weakest X→p(π) π-donation while mesoionic carbenes possess the strongest π-donation to the carbene centre. There is a reasonable correlation between the occupation of the p(π) AO at the C_carb_ atom and the energy which is associated with the X→p(π) π-donation.

## Supporting Information

File 1 Additional information.

## References

[1] Arduengo A J, Harlow R L, Kline M (1991). J Am Chem Soc.

[2] Bourissou D, Guerret O, Gabbaï F P, Bertrand G (2000). Chem Rev.

[3] Schuster O, Yang L, Raubenheimer H G, Albrecht M (2009). Chem Rev.

[4] Hopkinson M N, Richter C, Schedler M, Glorius F (2014). Nature.

[5] Benhamou L, Chardon E, Lavigne G, Bellemin-Laponnaz S, César V (2011). Chem Rev.

[6] Martin D, Melaimi M, Soleilhavoup M, Bertrand G (2011). Organometallics.

[7] Melaimi M, Soleilhavoup M, Bertrand G (2010). Angew Chem, Int Ed.

[8] Canac Y, Soleilhavoup M, Conejero S, Bertrand G (2004). J Organomet Chem.

[9] Díez-González S, Marion N, Nolan S P (2009). Chem Rev.

[10] Levin E, Ivry E, Diesendruck C E, Lemcoff N G (2015). Chem Rev.

[11] Schaper L-A, Hock S J, Herrmann W A, Kühn F E (2013). Angew Chem, Int Ed.

[12] Izquierdo J, Hutson G E, Cohen D T, Scheidt K A (2012). Angew Chem, Int Ed.

[13] Radius U, Bickelhaupt F M (2009). Coord Chem Rev.

[14] Fèvre M, Pinaud J, Gnanou Y, Vignolle J, Taton D (2013). Chem Soc Rev.

[15] Enders D, Niemeier O, Henseler A (2007). Chem Rev.

[16] Marion N, Díez-González S, Nolan S P (2007). Angew Chem, Int Ed.

[17] Hahn F E, Jahnke M C (2008). Angew Chem, Int Ed.

[18] Zhang D, Zi G (2015). Chem Soc Rev.

[19] Nelson D J (2015). Eur J Inorg Chem.

[20] Soleilhavoup M, Bertrand G (2015). Acc Chem Res.

[21] Wang Y, Robinson G H (2014). Inorg Chem.

[22] Wilson D J D, Dutton J L (2013). Chem – Eur J.

[23] Wang Y, Robinson G H (2012). Dalton Trans.

[24] Wang Y, Robinson G H (2011). Inorg Chem.

[25] Rivard E (2014). Dalton Trans.

[26] Martin C D, Soleilhavoup M, Bertrand G (2013). Chem Sci.

[27] Martin D, Soleilhavoup M, Bertrand G (2011). Chem Sci.

[28] Power P P (2010). Nature.

[29] de Frémont P, Marion N, Nolan S P (2009). Coord Chem Rev.

[30] Regitz M (1991). Angew Chem, Int Ed Engl.

[31] Arduengo A J, Rasika Dias H V, Harlow R L, Kline M (1992). J Am Chem Soc.

[32] Arduengo A J (1999). Acc Chem Res.

[33] Herrmann W A (2002). Angew Chem, Int Ed.

[34] Jacobsen H, Correa A, Poater A, Costabile C, Cavallo L (2009). Coord Chem Rev.

[35] Jacobsen H, Correa A, Costabile C, Cavallo L (2006). J Organomet Chem.

[36] Nemcsok D, Wichmann K, Frenking G (2004). Organometallics.

[37] Tonner R, Heydenrych G, Frenking G (2007). Chem – Asian J.

[38] Hahn F E, Zabula A V, Pape T, Hepp A, Tonner R, Haunschild R, Frenking G (2008). Chem – Eur J.

[39] Scarborough C C, Grady M J W, Guzei I A, Gandhi B A, Bunel E E, Stahl S S (2005). Angew Chem, Int Ed.

[40] Iglesias M, Beetstra D J, Kariuki B, Cavell K J, Dervisi A, Fallis I A (2009). Eur J Inorg Chem.

[41] Lu W Y, Cavell K J, Wixey J S, Kariuki B (2011). Organometallics.

[42] Martin D, Lassauque N, Donnadieu B, Bertrand G (2012). Angew Chem, Int Ed.

[43] Martin D, Lassauque N, Steinmann F, Manuel G, Bertrand G (2013). Chem – Eur J.

[44] Lavallo V, Dyker C A, Donnadieu B, Bertrand G (2008). Angew Chem, Int Ed.

[45] Fernández I, Dyker C A, DeHope A, Donnadieu B, Frenking G, Bertrand G (2009). J Am Chem Soc.

[46] Aldeco-Perez E, Rosenthal A J, Donnadieu B, Parameswaran P, Frenking G, Bertrand G (2009). Science.

[47] Guisado-Barrios G, Bouffard J, Donnadieu B, Bertrand G (2010). Angew Chem, Int Ed.

[48] Borthakur B, Phukan A K (2015). Chem – Eur J.

[49] Nakafuji S-y, Kobayashi J, Kawashima T (2008). Angew Chem, Int Ed.

[50] Asay M, Donnadieu B, Baceiredo A, Soleilhavoup M, Bertrand G (2008). Inorg Chem.

[51] Fürstner A, Alcarazo M, Radkowski K, Lehmann C W (2008). Angew Chem, Int Ed.

[52] Hudnall T W, Bielawski C W (2009). J Am Chem Soc.

[53] Hudnall T W, Tennyson A G, Bielawski C W (2010). Organometallics.

[54] César V, Lugan N, Lavigne G (2010). Chem – Eur J.

[55] Braun M, Frank W, Reiss G J, Ganter C (2010). Organometallics.

[56] Lavallo V, Canac Y, Präsang C, Donnadieu B, Bertrand G (2005). Angew Chem, Int Ed.

[57] Jazzar R, Dewhurst R D, Bourg J-B, Donnadieu B, Canac Y, Bertrand G (2007). Angew Chem, Int Ed.

[58] Díez-González S, Nolan S P (2007). Coord Chem Rev.

[59] Curran D P, Solovyev A, Makhlouf Brahmi M, Fensterbank L, Malacria M, Lacôte E (2011). Angew Chem, Int Ed.

[60] Frenking G, Tonner R, Klein S, Takagi N, Shimizu T, Krapp A, Pandey K K, Parameswaran P (2014). Chem Soc Rev.

[61] Dröge T, Glorius F (2010). Angew Chem, Int Ed.

[62] Nelson D J, Nolan S P (2013). Chem Soc Rev.

[63] Fantasia S, Petersen J L, Jacobsen H, Cavallo L, Nolan S P (2007). Organometallics.

[64] Back O, Henry-Ellinger M, Martin C D, Martin D, Bertrand G (2013). Angew Chem, Int Ed.

[65] Liske A, Verlinden K, Buhl H, Schaper K, Ganter C (2013). Organometallics.

[66] Vummaleti S V C, Nelson D J, Poater A, Gómez-Suárez A, Cordes D B, Slawin A M Z, Nolan S P, Cavallo L (2015). Chem Sci.

[67] Alcarazo M, Stork T, Anoop A, Thiel W, Fürstner A (2010). Angew Chem, Int Ed.

[68] Tukov A A, Normand A T, Nechaev M S (2009). Dalton Trans.

[69] Bernhammer J C, Frison G, Huynh H V (2013). Chem – Eur J.

[70] Huynh H V, Frison G (2013). J Org Chem.

[71] Comas-Vives A, Harvey J N (2011). Eur J Inorg Chem.

[72] Rezabal E, Frison G (2015). J Comput Chem.

[73] Crabtree R H (2013). Coord Chem Rev.

[74] Becke A D (1988). Phys Rev A.

[75] Perdew J P (1986). Phys Rev B.

[76] Weigend F, Ahlrichs R (2005). Phys Chem Chem Phys.

[77] Peng C, Ayala P Y, Schlegel H B, Frisch M J (1996). J Comput Chem.

[78] McIver J W, Komornicki A (1972). J Am Chem Soc.

[79] (2009). Gaussian 09.

[80] Wiberg K B (1968). Tetrahedron.

[81] Reed A E, Weinstock R B, Weinhold F (1985). J Chem Phys.

[82] Reed A E, Curtiss L A, Weinhold F (1988). Chem Rev.

[83] (2009). GENNBO.

[84] Van Lenthe E, Baerends E J (2003). J Comput Chem.

[R85] 85Krijn, J.; Baerends, E. J. *Fit Functions in the HFS-Method*; 1984.

[86] Van Lenthe E, Baerends E J, Snijders J G (1993). J Chem Phys.

[87] te Velde G, Bickelhaupt F M, Baerends E J, Fonseca Guerra C, van Gisbergen S J A, Snijders J G, Ziegler T (2001). J Comput Chem.

[88] Morokuma K (1971). J Chem Phys.

[89] Ziegler T, Rauk A (1979). Inorg Chem.

[90] Ziegler T, Rauk A (1979). Inorg Chem.

[91] Bickelhaupt F M, Nibbering N M M, Van Wezenbeek E M, Baerends E J (1992). J Phys Chem.

[92] Frenking G, Wichmann K, Fröhlich N, Loschen C, Lein M, Frunzke J, Rayón V M (2003). Coord Chem Rev.

[93] Krapp A, Bickelhaupt F M, Frenking G (2006). Chem – Eur J.

[94] Kovács A, Esterhuysen C, Frenking G (2005). Chem – Eur J.

[95] Mitoraj M P, Michalak A, Ziegler T (2009). J Chem Theory Comput.

[96] Mitoraj M, Michalak A (2007). Organometallics.

[97] Michalak A, Mitoraj M, Ziegler T (2008). J Phys Chem A.

[98] Mitoraj M, Michalak A (2008). J Mol Model.

[99] Hahn F E, Wittenbecher L, Boese R, Bläser D (1999). Chem – Eur J.

[100] Arduengo A J, Goerlich J R, Marshall W J (1995). J Am Chem Soc.

[101] Alder R W, Allen P R, Murray M, Orpen A G (1996). Angew Chem, Int Ed Engl.

[102] Korotkikh N I, Rayenko G F, Shvaika O P, Pekhtereva T M, Cowley A H, Jones J N, Macdonald C L B (2003). J Org Chem.

[103] Arduengo A J, Goerlich J R, Marshall W J (1997). Liebigs Ann/Recl.

[104] Pyykkö P, Atsumi M (2009). Chem – Eur J.

[105] Heinemann C, Müller T, Apeloig Y, Schwarz H (1996). J Am Chem Soc.

[106] Tuononen H M, Roesler R, Dutton J L, Ragogna P J (2007). Inorg Chem.

[107] Boehme C, Frenking G (1996). J Am Chem Soc.

[108] Mondal K C, Roesky H W, Schwarzer M C, Frenking G, Niepötter B, Wolf H, Herbst-Irmer R, Stalke D (2013). Angew Chem, Int Ed.

[109] Mondal K C, Roesky H W, Schwarzer M C, Frenking G, Tkach I, Wolf H, Kratzert D, Herbst-Irmer R, Niepötter B, Stalke D (2013). Angew Chem, Int Ed.

[110] Singh A P, Samuel P P, Roesky H W, Schwarzer M C, Frenking G, Sidhu N S, Dittrich B (2013). J Am Chem Soc.

[111] Weinberger D S, Melaimi M, Moore C E, Rheingold A L, Frenking G, Jerabek P, Bertrand G (2013). Angew Chem, Int Ed.

[112] Samuel P P, Mondal K C, Roesky H W, Hermann M, Frenking G, Demeshko S, Meyer F, Stückl A C, Christian J H, Dalal N S (2013). Angew Chem, Int Ed.

[113] Mondal K C, Samuel P P, Roesky H W, Carl E, Herbst-Irmer R, Stalke D, Schwederski B, Kaim W, Ungur L, Chibotaru L F (2014). J Am Chem Soc.

[114] Weinberger D S, Amin Sk N, Mondal K C, Melaimi M, Bertrand G, Stückl A C, Roesky H W, Dittrich B, Demeshko S, Schwederski B (2014). J Am Chem Soc.

[115] Mondal K C, Samuel P P, Roesky H W, Aysin R R, Leites L A, Neudeck S, Lübben J, Dittrich B, Holzmann N, Hermann M (2014). J Am Chem Soc.

[116] Roy S, Mondal K C, Meyer J, Niepötter B, Köhler C, Herbst-Irmer R, Stalke D, Dittrich B, Andrada D M, Frenking G (2015). Chem – Eur J.

[117] Roy S, Stollberg P, Herbst-Irmer R, Stalke D, Andrada D M, Frenking G, Roesky H W (2015). J Am Chem Soc.

[R118] 118The molecules **4**, **5** and **9** were calculated for technical reasons with enforce *C*_s_ symmetry in order to align the single occupied orbitals in the right way. The energy differences to the fully optimized structures are negligible and thus, the results for the structures with *C*_s_ symmetry can be used for the bonding analysis of the equilibrium structures.

[119] Esterhuysen C, Frenking G (2004). Theor Chem Acc.

[120] Frenking G, Wichmann K, Fröhlich N, Grobe J, Golla W, Le Van D, Krebs B, Läge M (2002). Organometallics.

