# Supplementary material for: Direct estimate of the internal π-donation to the carbene centre within N-heterocyclic carbenes and related molecules
Source: Beilstein J Org Chem. 2015 Dec 24;11:2727–36. doi: 10.3762/bjoc.11.294 (PMC4734353; doi:10.3762/bjoc.11.294)
Supplement: File 1 — Additional information. [file Beilstein_J_Org_Chem-11-2727-s001.pdf]

Supporting Information

for

Direct estimate of the internal π-donation to the carbene centre within N-heterocyclic carbenes and related molecules

Diego M. Andrada[1], Nicole Holzmann[2], Thomas Hamadi[1] and Gernot Frenking[1,*,§]

Address: [1]Fachbereich Chemie, Philipps-Universität Marburg, Hans-Meerwein-Strasse, D-35032 Marburg, Germany and [2]Laboratoire International Associé Centre National de la Recherche Scientifique - UMR 7565, Université de Lorraine, 54506 Vandoeuvre-lès-Nancy, France

[§]Fax: +49-6421-2825566

Email: Gernot Frenking - frenking@chemie.uni-marburg.de

Additional information

Contents:

Table S1. Vertical singlet-triplet energy gap and single-triplet gap [in kcal/mol] at BP86/def2-TZVPP.

Table S2. Cartesian xyz coordinates (in Å) and electronic energies (in Hartree) of the calculated structures at BP86/def2-TZVPP.

Figure S1. Plot of deformation densities Δρ of the pairwise orbital interactions on the carbenes **2–15**, associated energies ΔE in kcal/mol and eigenvalues ν in a.u.

2

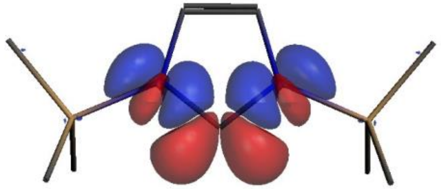

ΔE_1=-331.0; $|\nu|_{\alpha} = 0.70$; $|\nu|_{\beta} = 0.67$

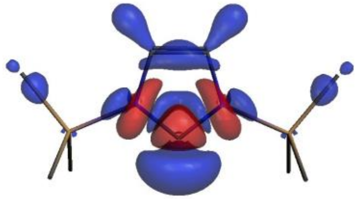

ΔE_2=-247.2; $|\nu|_{\alpha} = 0.65$; $|\nu|_{\beta} = 0.37$

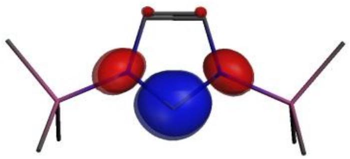

ΔE_3=-87.8 ; $|\nu|_{\alpha} = 0.46$; $|\nu|_{\beta} = 0.46$

3

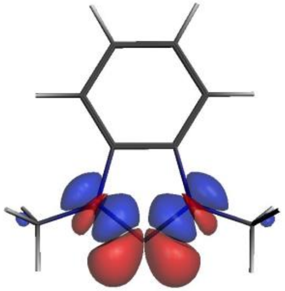

ΔE_1=-322.1; $|\nu|_{\alpha} = 0.68$; $|\nu|_{\beta} = 0.64$

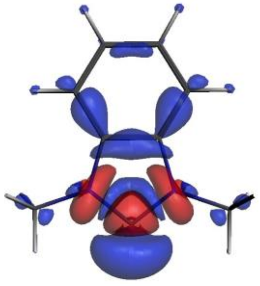

ΔE_2=-252.2; $|\nu|_{\alpha} = 0.65$; $|\nu|_{\beta} = 0.36$

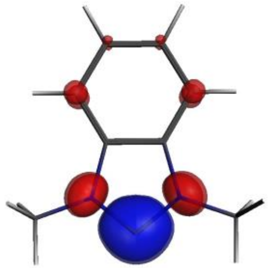

ΔE_3=-89.3 ; $|\nu|_{\alpha} = 0.46$; $|\nu|_{\beta} = 0.46$

4

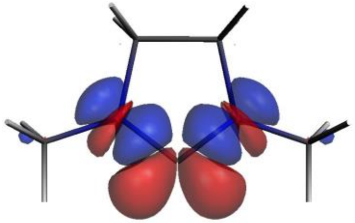

ΔE_1=-335.9; $|\nu|_{\alpha} = 0.66$; $|\nu|_{\beta} = 0.66$

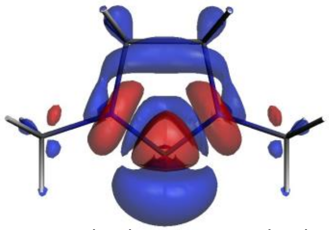

ΔE_2=-242.3; $|\nu|_{\alpha} = 0.60$; $|\nu|_{\beta} = 0.37$

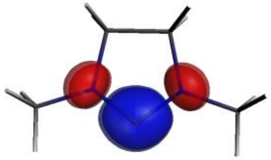

ΔE_3=-86.1; $|\nu|_{\alpha} = 0.41$; $|\nu|_{\beta} = 0.41$

5

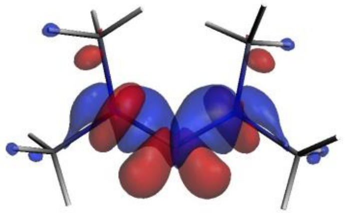

ΔE_1=-379.5; $|\nu|_\alpha = 0.73$; $|\nu|_\beta = 0.69$

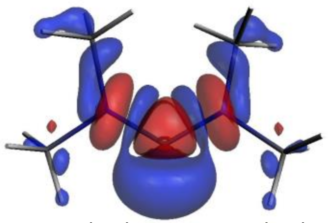

ΔE_2=-335.4; $|\nu|_\alpha = 0.60$; $|\nu|_\beta = 0.37$

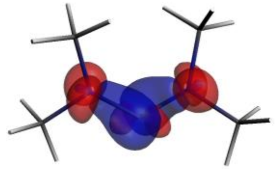

ΔE_3=-84.3; $|\nu|_\alpha = 0.38$; $|\nu|_\beta = 0.42$

6

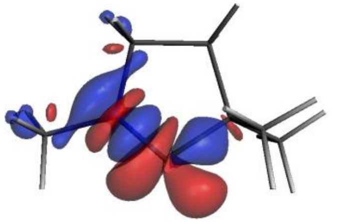

ΔE_1=-331.7; $|\nu|_\alpha = 0.64$; $|\nu|_\beta = 0.70$

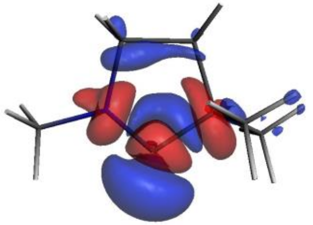

ΔE_2=-182.6; $|\nu|_\alpha = 0.54$; $|\nu|_\beta = 0.41$

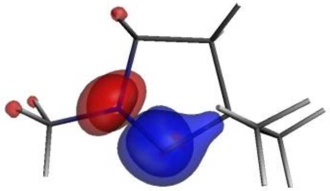

ΔE_3=-72.2; $|\nu|_\alpha = 0.38$; $|\nu|_\beta = 0.38$

7

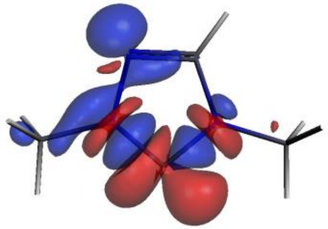

ΔE_1=-338.7; $|\nu|_\alpha = 0.69$; $|\nu|_\beta = 0.63$

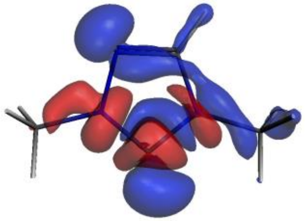

ΔE_2=-241.7; $|\nu|_\alpha = 0.68$; $|\nu|_\beta = 0.36$

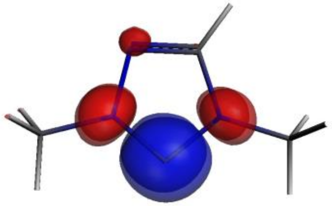

ΔE_3=-92.2; $|\nu|_\alpha = 0.44$; $|\nu|_\beta = 0.44$

8

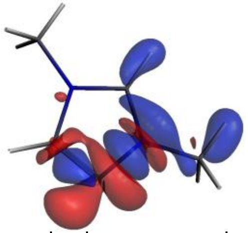

ΔE_1=-327.6; $|\nu|_\alpha = 0.67$; $|\nu|_\beta = 0.67$

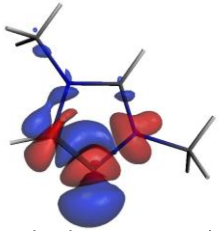

ΔE_2=-170.8; $|\nu|_\alpha = 0.48$; $|\nu|_\beta = 0.37$

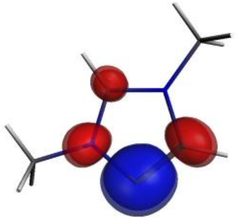

ΔE_3=-101.3; $|\nu|_\alpha = 0.47$; $|\nu|_\beta = 0.47$

9

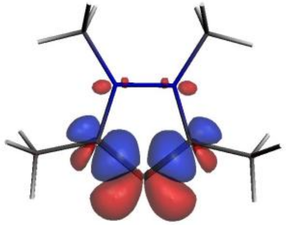

ΔE_1=-285.9; $|\nu|_{\alpha}$ = 0.55; $|\nu|_{\beta}$ = 0.68

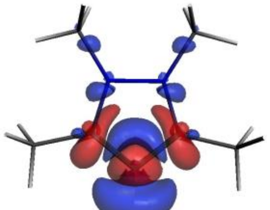

ΔE_2=-178.5; $|\nu|_{\alpha}$ = 0.46; $|\nu|_{\beta}$ = 0.42

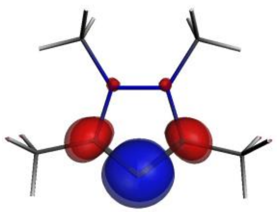

ΔE_3=-109.9; $|\nu|_{\alpha}$ = 0.45; $|\nu|_{\beta}$ = 0.45

10

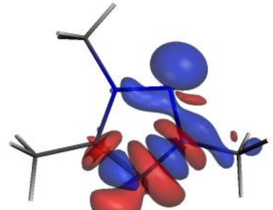

ΔE_1=-335.8; $|\nu|_{\alpha}$ = 0.69; $|\nu|_{\beta}$ = 0.67

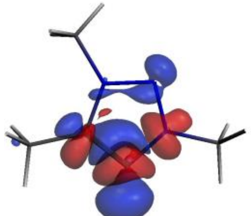

ΔE_2=-186.6; $|\nu|_{\alpha}$ = 0.52; $|\nu|_{\beta}$ = 0.38

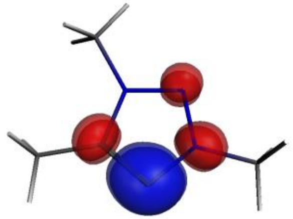

ΔE_3=-100.6; $|\nu|_{\alpha}$ = 0.46; $|\nu|_{\beta}$ = 0.46

11

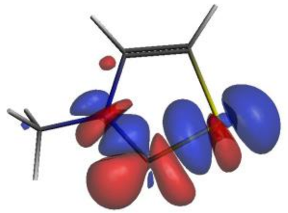

ΔE_1=-256.9; $|\nu|_{\alpha}$ = 0.67; $|\nu|_{\beta}$ = 0.67

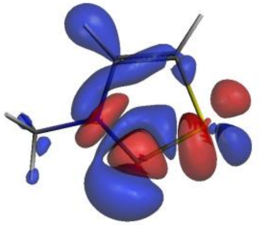

ΔE_2=-219.0; $|\nu|_{\alpha}$ = 0.62; $|\nu|_{\beta}$ = 0.39

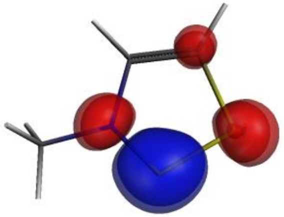

ΔE_3=-90.0; $|\nu|_{\alpha}$ = 0.47; $|\nu|_{\beta}$ = 0.47

12

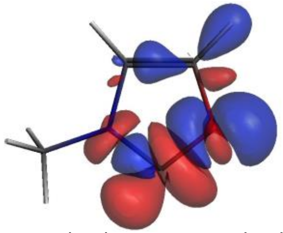

ΔE_1=-361.0; $|\nu|_{\alpha}$ = 0.83; $|\nu|_{\beta}$ = 0.61

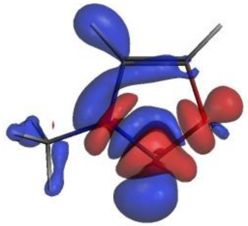

ΔE_2=-235.7; $|\nu|_{\alpha}$ = 0.64; $|\nu|_{\beta}$ = 0.34

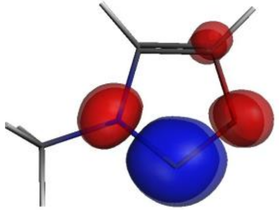

ΔE_3=-82.4; $|\nu|_{\alpha}$ = 0.44; $|\nu|_{\beta}$ = 0.44

13

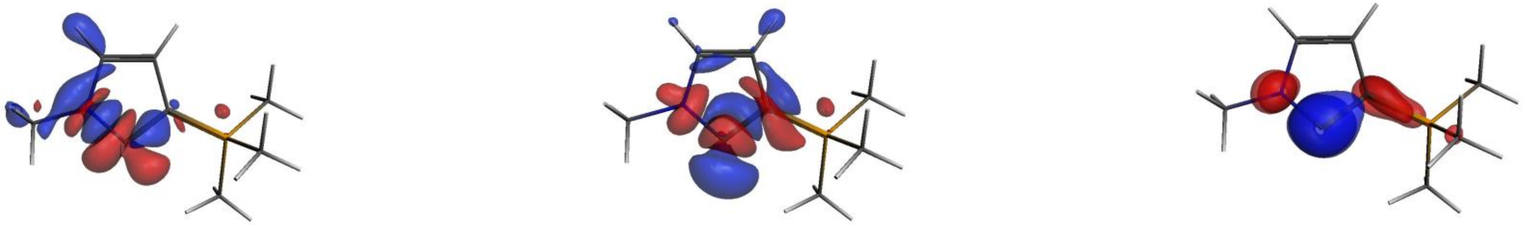

ΔE_1=-326.8; $|\nu|_\alpha$ = 0.67; $|\nu|_\beta$ = 0.72 ΔE_2=-180.9; $|\nu|_\alpha$ = 0.52; $|\nu|_\beta$ = 0.40 ΔE_3=-96.4; $|\nu|_\alpha$ = 0.47; $|\nu|_\beta$ = 0.47

14

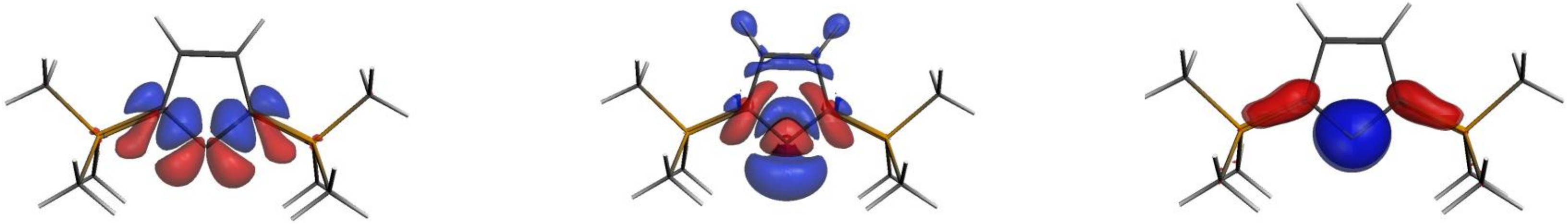

ΔE_1=-283.0; $|\nu|_\alpha$ = 0.56; $|\nu|_\beta$ = 0.76 ΔE_2=-184.7; $|\nu|_\alpha$ = 0.49; $|\nu|_\beta$ = 0.46 ΔE_3=-100.8; $|\nu|_\alpha$ = 0.51; $|\nu|_\beta$ = 0.51

15

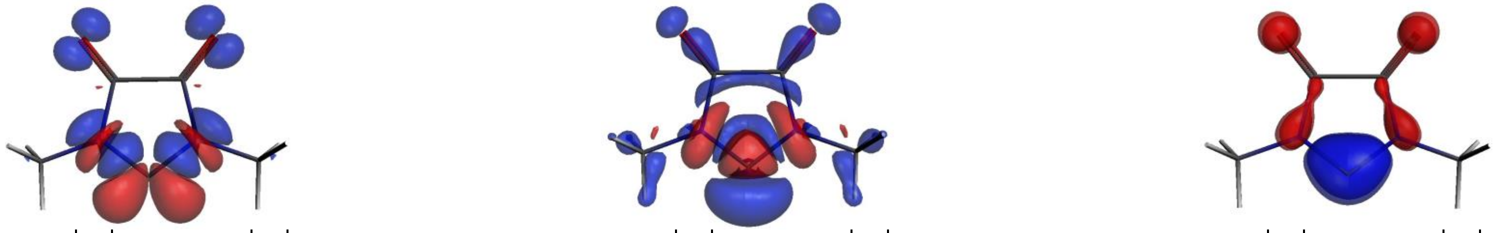

ΔE_1=-322.0; $|\nu|_\alpha$ = 0.70; $|\nu|_\beta$ = 0.65 ΔE_2=-242.0; $|\nu|_\alpha$ = 0.64; $|\nu|_\beta$ = 0.36 ΔE_3=-74.0; $|\nu|_\alpha$ = 0.42; $|\nu|_\beta$ = 0.42

Figure S1. Plot of deformation densities $\Delta\rho$ of the pairwise orbital interactions on the carbenes **2-15**, associated energies ΔE in kcal/mol and eigenvalues ν in a.u. The color-code is red →blue for the charge flow.

Table S1. Vertical singlet-triplet energy gap and single-triplet gap [in kcal/mol] at BP86/def2-TZVPP.

	$\Delta E_{(S\text{-}T)}^{vert}$	$\Delta E_{(S\text{-}T)}$
**1**	99.1	91.6
**2**	89.7	77.5
**3**	82.8	78.4
**4**	90.9	72.9
**5**	77.0	42.2
**6**	63.5	49.3
**7**	99.1	83.3
**8**	86.5	59.6
**9**	83.8	40.7
**10**	83.3	60.0
**11**	75.6	66.2
**12**	90.9	83.3
**13**	85.1	61.4
**14**	74.6	54.1
**15**	70.9	27.5

Table S2. Cartesian xyz coordinates (in Å) and electronic energies (in Hartree) of the calculated structures at BP86/def2-TZVPP.

1

E = -304.9123197

C	-0.000002000000	0.986769000000	-0.000011000000
N	1.065462000000	0.120004000000	-0.000053000000
N	-1.065460000000	0.120011000000	0.000051000000
C	0.681552000000	-1.218447000000	-0.000031000000
C	-0.681552000000	-1.218454000000	0.000031000000
C	2.447729000000	0.574822000000	0.000025000000
H	1.386178000000	-2.043307000000	-0.000064000000
H	-1.386186000000	-2.043305000000	0.000063000000
C	-2.447728000000	0.574821000000	-0.000020000000
H	2.977385000000	0.217990000000	0.895462000000
H	2.977752000000	0.217084000000	-0.894826000000
H	2.431812000000	1.669652000000	-0.000507000000
H	-2.977374000000	0.218027000000	-0.895476000000
H	-2.977756000000	0.217038000000	0.894813000000
H	-2.431829000000	1.669650000000	0.000585000000

2

E= -1043.8187311

C	0.000012000000	-0.382471000000	-0.000033000000
C	-0.679938000000	1.813875000000	0.000014000000
C	0.679943000000	1.813879000000	0.000032000000
N	-1.084518000000	0.471065000000	-0.000034000000
N	1.084535000000	0.471075000000	0.000004000000
H	-1.376884000000	2.644797000000	-0.000018000000
H	1.376880000000	2.644809000000	0.000046000000
Si	-2.738663000000	-0.235021000000	0.000019000000
Si	2.738678000000	-0.235019000000	-0.000004000000
C	-2.929786000000	-1.272686000000	-1.551120000000
H	-3.917875000000	-1.755989000000	-1.587475000000
H	-2.816651000000	-0.660440000000	-2.457602000000
H	-2.158326000000	-2.054864000000	-1.572709000000
C	-2.929621000000	-1.272840000000	1.551067000000
H	-3.917975000000	-1.755535000000	1.587972000000
H	-2.158578000000	-2.055446000000	1.572084000000
H	-2.815516000000	-0.660777000000	2.457556000000
C	-3.941600000000	1.214867000000	0.000085000000
H	-3.822196000000	1.848665000000	-0.890932000000
H	-4.976937000000	0.841792000000	0.000288000000
H	-3.821925000000	1.848841000000	0.890943000000
C	2.929583000000	-1.273111000000	1.550867000000
H	2.158626000000	-2.055806000000	1.571669000000
H	3.917987000000	-1.755707000000	1.587769000000
H	2.815333000000	-0.661230000000	2.457460000000
C	2.929783000000	-1.272449000000	-1.551304000000
H	2.158207000000	-2.054512000000	-1.573006000000
H	2.816736000000	-0.660065000000	-2.457703000000
H	3.917801000000	-1.755888000000	-1.587711000000
C	3.941608000000	1.214871000000	0.000387000000
H	4.976953000000	0.841820000000	0.000073000000
H	3.821918000000	1.849117000000	-0.890273000000
H	3.822188000000	1.848392000000	0.891602000000

3

E= -458.6160457

C	0.000000000000	0.000000000000	2.026913000000
C	0.000000000000	0.703268000000	-2.559152000000
C	0.000000000000	1.431684000000	-1.364398000000
C	0.000000000000	-0.703268000000	-2.559152000000
H	0.000000000000	2.522127000000	-1.369609000000
H	0.000000000000	-1.236881000000	-3.510446000000
C	0.000000000000	0.705470000000	-0.171217000000
C	0.000000000000	-1.431684000000	-1.364398000000
H	0.000000000000	-2.522127000000	-1.369609000000
C	0.000000000000	-0.705470000000	-0.171217000000
H	0.000000000000	1.236881000000	-3.510446000000
N	0.000000000000	-1.078214000000	1.173600000000
N	0.000000000000	1.078214000000	1.173600000000
C	0.000000000000	-2.454103000000	1.640761000000
H	0.000000000000	-2.428794000000	2.735079000000
H	-0.894670000000	-2.987652000000	1.286536000000
H	0.894670000000	-2.987652000000	1.286536000000
C	0.000000000000	2.454103000000	1.640761000000
H	-0.894670000000	2.987652000000	1.286536000000
H	0.000000000000	2.428794000000	2.735079000000
H	0.894670000000	2.987652000000	1.286536000000

4

E= -306.1130532

C	-0.000018000000	-1.023178000000	-0.000001000000
N	-1.076249000000	-0.210399000000	-0.038830000000
N	1.076226000000	-0.210411000000	0.038969000000
C	-0.767690000000	1.240147000000	0.033444000000
C	0.767762000000	1.240136000000	-0.033474000000
C	-2.447312000000	-0.663991000000	-0.001924000000
H	-1.236736000000	1.785980000000	-0.800182000000
H	1.153171000000	1.669485000000	-0.974471000000
C	2.447281000000	-0.664022000000	0.001871000000
H	-2.954444000000	-0.343313000000	0.925867000000
H	-3.022100000000	-0.268222000000	-0.856841000000
H	-2.444562000000	-1.758455000000	-0.046457000000
H	2.954358000000	-0.342935000000	-0.925812000000
H	3.022144000000	-0.268620000000	0.856914000000
H	2.444552000000	-1.758504000000	0.045952000000
H	1.236671000000	1.786035000000	0.800180000000
H	-1.153035000000	1.669660000000	0.974380000000

5

E= -307.2971988

C	0.000019000000	-0.814852000000	-0.000004000000
N	-1.170947000000	-0.139636000000	-0.006927000000
N	1.170967000000	-0.139647000000	0.006875000000
C	1.470671000000	1.273594000000	-0.301421000000
H	1.652343000000	1.882896000000	0.600165000000
H	2.384805000000	1.308315000000	-0.913811000000
H	0.661291000000	1.722865000000	-0.883464000000
C	2.378275000000	-0.945177000000	0.186188000000
H	2.070544000000	-1.985908000000	0.326253000000
H	3.039837000000	-0.865584000000	-0.694851000000
H	2.947052000000	-0.610711000000	1.070676000000
C	-2.378241000000	-0.945194000000	-0.186173000000
H	-2.947238000000	-0.610517000000	-1.070442000000
H	-2.070505000000	-1.985875000000	-0.326565000000
H	-3.039628000000	-0.865875000000	0.695030000000

C	-1.470718000000	1.273532000000	0.301435000000
H	-2.384660000000	1.308179000000	0.914136000000
H	-0.661238000000	1.722973000000	0.883195000000
H	-1.652783000000	1.882794000000	-0.600108000000

6

E= -329.3730921

C	-0.227423000000	-1.024142000000	-0.111393000000
N	-1.268922000000	-0.229454000000	-0.062664000000
C	-1.023724000000	1.248888000000	0.043436000000
C	0.468795000000	1.341806000000	-0.268363000000
C	0.998105000000	-0.113880000000	-0.024036000000
H	-1.669754000000	1.790152000000	-0.664000000000
H	-1.286891000000	1.584592000000	1.060350000000
H	0.983389000000	2.090892000000	0.349247000000
H	0.618671000000	1.621349000000	-1.321723000000
C	-2.652869000000	-0.692860000000	-0.040339000000
H	-3.212820000000	-0.266075000000	-0.886936000000
H	-2.646241000000	-1.785074000000	-0.106625000000
H	-3.145834000000	-0.377629000000	0.892722000000
C	2.064059000000	-0.524740000000	-1.049370000000
H	2.396996000000	-1.555841000000	-0.866913000000
H	1.667466000000	-0.477320000000	-2.074314000000
H	2.939952000000	0.140776000000	-0.989599000000
C	1.560128000000	-0.272146000000	1.407343000000
H	0.835293000000	0.055687000000	2.168022000000
H	1.805563000000	-1.323492000000	1.611013000000
H	2.474240000000	0.330605000000	1.523732000000

7

E= -320.9616574

C	-0.002624000000	-1.004149000000	-0.000018000000
N	1.057836000000	-0.118874000000	0.000010000000
N	-1.041946000000	-0.135115000000	-0.000019000000
C	0.596852000000	1.180467000000	0.000051000000
N	-0.711673000000	1.214605000000	0.000047000000
C	2.456731000000	-0.526311000000	-0.000065000000
H	1.229353000000	2.063154000000	0.000078000000
C	-2.448872000000	-0.497194000000	0.000008000000
H	2.972130000000	-0.152912000000	-0.895861000000
H	2.972408000000	-0.152211000000	0.895276000000
H	2.477984000000	-1.620573000000	0.000351000000
H	-2.943422000000	-0.094771000000	0.894026000000
H	-2.943597000000	-0.094112000000	-0.893612000000
H	-2.506904000000	-1.589759000000	-0.000376000000

8

E=-304.8849308

C	-0.028675000000	-0.848518000000	0.000018000000
N	1.068662000000	-0.071926000000	0.000043000000
N	-1.096827000000	-0.021738000000	-0.000033000000
C	0.634828000000	1.260930000000	0.000021000000
C	-0.751322000000	1.345926000000	-0.000004000000
C	2.446613000000	-0.548793000000	-0.000042000000
H	1.378743000000	2.052635000000	0.000035000000
C	-2.479267000000	-0.486423000000	0.000005000000
H	2.971515000000	-0.185722000000	-0.894005000000
H	2.971867000000	-0.184868000000	0.893363000000
H	2.462147000000	-1.644656000000	0.000488000000
H	-2.992227000000	-0.101706000000	0.890139000000

H	-2.992528000000	-0.100738000000	-0.889528000000
H	-2.512087000000	-1.584185000000	-0.000576000000
H	-0.023335000000	-1.933838000000	0.000023000000

9

E=-383.5049069

C	0.000000000000	1.687091000000	0.000001000000
C	-1.089701000000	0.802531000000	0.031315000000
C	1.089701000000	0.802531000000	-0.031315000000
N	-0.685939000000	-0.521545000000	0.034914000000
N	0.685939000000	-0.521545000000	-0.034913000000
C	-2.552667000000	1.126598000000	0.055827000000
C	2.552667000000	1.126598000000	-0.055828000000
H	-3.075043000000	0.798754000000	-0.858622000000
H	-3.076605000000	0.680136000000	0.917221000000
H	-2.644006000000	2.216084000000	0.124438000000
H	3.075047000000	0.798746000000	0.858616000000
H	3.076603000000	0.680145000000	-0.917228000000
H	2.644005000000	2.216085000000	-0.124429000000
C	1.445258000000	-1.749267000000	0.120350000000
H	1.313997000000	-2.191696000000	1.121578000000
H	1.171967000000	-2.495362000000	-0.640928000000
H	2.502387000000	-1.497544000000	-0.011247000000
C	-1.445258000000	-1.749267000000	-0.120350000000
H	-1.313996000000	-2.191695000000	-1.121578000000
H	-1.171967000000	-2.495362000000	0.640928000000
H	-2.502387000000	-1.497544000000	0.011246000000

10

E=-360.2530547

C	-0.584167000000	1.293713000000	0.000192000000
C	0.710663000000	0.741827000000	-0.000187000000
N	-1.327257000000	0.135024000000	0.000449000000
N	0.604040000000	-0.631439000000	-0.000172000000
N	-0.662044000000	-1.039072000000	0.000191000000
C	2.034240000000	1.432054000000	-0.000063000000
C	-2.784511000000	0.061750000000	-0.000246000000
H	2.636664000000	1.190016000000	0.890719000000
H	2.640725000000	1.182887000000	-0.886071000000
H	1.855573000000	2.513181000000	-0.004659000000
H	-3.139069000000	-0.458671000000	-0.899580000000
H	-3.138945000000	-0.474056000000	0.889987000000
H	-3.149738000000	1.091999000000	0.008274000000
C	1.660823000000	-1.634472000000	-0.000020000000
H	2.287676000000	-1.521367000000	0.894437000000
H	1.193607000000	-2.623636000000	-0.000244000000
H	2.288044000000	-1.521175000000	-0.894195000000

11

E=-608.4542923

C	-0.087560000000	-1.163656000000	0.000080000000
N	-0.901659000000	-0.076941000000	0.000070000000
S	1.511666000000	-0.524981000000	-0.000026000000
C	1.030316000000	1.151400000000	-0.000033000000
C	-0.325750000000	1.198736000000	0.000056000000
H	1.717463000000	1.992136000000	-0.000077000000
H	-0.960230000000	2.082729000000	0.000084000000
C	-2.361326000000	-0.234667000000	-0.000066000000
H	-2.796229000000	0.230409000000	0.895653000000
H	-2.795986000000	0.229530000000	-0.896364000000

H	-2.574143000000	-1.307402000000	0.000412000000

12
E=-285.4502811

C	-0.090966000000	-1.193126000000	-0.000024000000
O	-1.399614000000	-0.764127000000	-0.000004000000
N	0.617230000000	-0.032391000000	-0.000013000000
C	-0.209051000000	1.100013000000	-0.000045000000
C	-1.467577000000	0.616569000000	0.000044000000
H	-2.446337000000	1.080411000000	0.000087000000
H	0.167850000000	2.115833000000	-0.000074000000
C	2.073444000000	0.001360000000	0.000027000000
H	2.423182000000	-1.036263000000	-0.000204000000
H	2.448240000000	0.515231000000	0.895974000000
H	2.448262000000	0.515646000000	-0.895671000000

13
E=-709.496093

C	-1.131388000000	-0.822136000000	-0.054880000000
N	-2.342798000000	-0.168569000000	0.008116000000
C	-2.236732000000	1.224482000000	0.026751000000
C	-0.902956000000	1.548873000000	-0.019069000000
C	-0.217692000000	0.278391000000	-0.084637000000
H	-3.112522000000	1.869484000000	0.054769000000
H	-0.493012000000	2.556838000000	-0.022248000000
P	1.465377000000	-0.091236000000	-0.012899000000
C	1.765556000000	-1.669564000000	-0.847674000000
H	0.912165000000	-2.305929000000	-0.562896000000
H	1.735401000000	-1.520094000000	-1.934148000000
H	2.724538000000	-2.114291000000	-0.551603000000
C	2.496361000000	1.202340000000	-0.777482000000
H	3.562306000000	0.947731000000	-0.709212000000
H	2.211128000000	1.314367000000	-1.831019000000
H	2.324442000000	2.157759000000	-0.264109000000
C	2.127435000000	-0.278072000000	1.682680000000
H	1.945105000000	0.648163000000	2.243540000000
H	1.575975000000	-1.092574000000	2.170583000000
H	3.203342000000	-0.503333000000	1.683187000000
C	-3.622553000000	-0.861192000000	0.031856000000
H	-4.223305000000	-0.621412000000	-0.859919000000
H	-3.411782000000	-1.935786000000	0.046367000000
H	-4.203038000000	-0.591127000000	0.928118000000

14
E=-1114.0756502

C	-0.000019000000	-0.401995000000	-0.144441000000
C	-1.098673000000	0.516880000000	-0.100076000000
C	-0.692522000000	1.906289000000	-0.002725000000
C	0.692585000000	1.906258000000	-0.002774000000
C	1.098681000000	0.516836000000	-0.100179000000
H	-1.330322000000	2.791582000000	0.022210000000
H	1.330423000000	2.791526000000	0.022098000000
P	2.672941000000	-0.179576000000	-0.005421000000
P	-2.672932000000	-0.179531000000	-0.005391000000
C	2.803289000000	-1.595547000000	-1.133363000000
H	1.856057000000	-2.141476000000	-1.000525000000
H	2.852946000000	-1.232331000000	-2.167770000000
H	3.670805000000	-2.231795000000	-0.911375000000
C	4.008439000000	1.001421000000	-0.398281000000
H	4.995457000000	0.527636000000	-0.313962000000

H	3.868948000000	1.379618000000	-1.418881000000
H	3.956702000000	1.848118000000	0.298925000000
C	3.107093000000	-0.847033000000	1.644508000000
H	3.078166000000	-0.030556000000	2.378206000000
H	2.333686000000	-1.580027000000	1.910358000000
H	4.098038000000	-1.323300000000	1.657427000000
C	-2.803477000000	-1.595307000000	-1.133561000000
H	-3.671304000000	-2.231238000000	-0.911858000000
H	-2.852744000000	-1.231940000000	-2.167932000000
H	-1.856517000000	-2.141654000000	-1.000596000000
C	-4.008458000000	1.001513000000	-0.398010000000
H	-3.956320000000	1.848424000000	0.298904000000
H	-3.869388000000	1.379373000000	-1.418792000000
H	-4.995486000000	0.527851000000	-0.313112000000
C	-3.106942000000	-0.847340000000	1.644429000000
H	-4.098051000000	-1.323258000000	1.657464000000
H	-2.333713000000	-1.580685000000	1.909842000000
H	-3.077502000000	-0.031112000000	2.378382000000

15

E=-454.2397744

C	0.000000000000	1.419870000000	-0.000004000000
N	-1.098447000000	0.586421000000	-0.000004000000
N	1.098448000000	0.586421000000	0.000002000000
C	-0.778690000000	-0.775505000000	-0.000017000000
C	0.778689000000	-0.775505000000	0.000019000000
C	-2.472165000000	1.077088000000	-0.000026000000
C	2.472165000000	1.077087000000	0.000025000000
H	-3.004237000000	0.720222000000	-0.891916000000
H	-3.004474000000	0.719553000000	0.891452000000
H	-2.429020000000	2.170758000000	0.000377000000
H	3.004221000000	0.720271000000	0.891946000000
H	3.004491000000	0.719501000000	-0.891422000000
H	2.429022000000	2.170756000000	-0.000437000000
O	1.531584000000	-1.723072000000	0.000030000000
O	-1.531585000000	-1.723073000000	-0.000025000000
